# A Computational Architecture Based on RFID Sensors for Traceability in Smart Cities

**DOI:** 10.3390/s150613591

**Published:** 2015-06-10

**Authors:** Higinio Mora-Mora, Virgilio Gilart-Iglesias, David Gil, Alejandro Sirvent-Llamas

**Affiliations:** Specialized Processors Architecture Laboratory, Department of Computer Science Technology and Computation, University of Alicante, 03690 Alicante, Spain; E-Mails: vgilart@ua.es (V.G.-I.); dgil@ua.es (D.G.); asirvent@ua.es (A.S.-L.)

**Keywords:** RFID smart sensor network, cyber physical systems, communication technology, computational architectures, smart city, track and trace system

## Abstract

Information Technology and Communications (ICT) is presented as the main element in order to achieve more efficient and sustainable city resource management, while making sure that the needs of the citizens to improve their quality of life are satisfied. A key element will be the creation of new systems that allow the acquisition of context information, automatically and transparently, in order to provide it to decision support systems. In this paper, we present a novel distributed system for obtaining, representing and providing the flow and movement of people in densely populated geographical areas. In order to accomplish these tasks, we propose the design of a smart sensor network based on RFID communication technologies, reliability patterns and integration techniques. Contrary to other proposals, this system represents a comprehensive solution that permits the acquisition of user information in a transparent and reliable way in a non-controlled and heterogeneous environment. This knowledge will be useful in moving towards the design of smart cities in which decision support on transport strategies, business evaluation or initiatives in the tourism sector will be supported by real relevant information. As a final result, a case study will be presented which will allow the validation of the proposal.

## 1. Introduction

Technological progress in recent years has made it possible to extend the use of Information Technology and Communications (ICT) to new applications with the objective of improving citizens’ quality of life. This idea has been of increasing significance in the political agenda as well as in the public services area. In the cases where there is no planning of development consistent with the efficient and sustainable management of urban resources this can start to cause real issues.

It is recognized that to either obtain a common definition of Smart City (SC) or to define global trends for SC is complicated. There are several reasons that contribute to this. On one hand, cities are complex systems typified by massive numbers of interconnected citizens, a lot of businesses, various modes of transport, communication networks, including services as well as utilities. On the other hand, this leads to a big diversity of technological, social, economic and organisational problems which expose the environmental sustainability of cities. Besides, there is no total consensus about the significance of SC but there is wide agreement concerning the fact that SCs are characterised by the use of ICTs. The goal is, in general, to make better use of resources in cities [1]. There are numerous examples which we could cite of cities that are supplementarily prepared with ICT systems in order to meet this objective in the fields of energy, telecommunications, ecology or transport [2,3,4,5]. Improvements in the management efficiency of these elements are directly perceived by citizens as quality of life improvements.

These reasons have led us to work on the citizen track and trace problem in urban environments. Progress on this issue may provide relevant information for decision-making in many of the above problems related to the management of a smart city, such as in the planning of transport infrastructure (roads, subway, bus lines) or modification of existing ones, in the construction of new public buildings (schools, hospitals, museums, sports areas), in designing tourist routes through the city, *etc.* Other uses of the obtained information can contemplate economic objectives such as a commercial rating of avenues and streets or the design of tourist offerings in the city.

Moreover, building increased intelligence into things around us and connecting them to the network to transfer data without requiring human interaction is a current trend of technological development called the Internet of Things (IoT) that allows us to meet and interact better with the surrounding environment [6,7]. IoT technologies such as sensing, computing and mobile communication could be employed to collect data on the functioning of the city. In this respect, how the data of interest is collected is the major challenge in most cases. Once obtained, another important issue is how to connect sensors to the web [8] to enable provision of cloud-based management and services. In this field, the development of distributed sensor infrastructure is one of the prominent research areas related to IoT [9,10].

In the work described in this paper, a method and a computer architecture to determine the movements of people in the city is proposed. The research done takes into account the current communication technologies, the ease of implementation and deployment and key aspects about user privacy. The main scientific contribution of this research is the integration of several new technologies and solutions of IoT to build a comprehensive system to track and trace citizens in city working environments. As a result, smart sensing technologies with processing and communication capabilities are proposed to produce cloud services for citizen traceability.

The remainder of the paper is organized as follows: Section 2 covers problem analysis, related work and the research methodology used; Section 3 presents the methods to know citizens’ movements with its dominant distributed components and decisions taken; Section 4 discusses aspects of communication and structuring of location information and describes the centralized architecture; Section 5 describes the main characteristics of the services provided for using the generated information; next, Section 6 describes the implementation based on a case study in a university environment to check the correct performance; finally Section 7 concludes with a discussion of critical outstanding issues and future research directions in this area.

## 2. Population Track and Trace Issues

### 2.1. Problem Definition

Track and trace is a term frequently used by delivery and logistics companies [11,12,13,14,15]. In the context of items and products, track and trace implies a process of determining their traceability understood as the current and past locations (or even more information) of a single item. In this work, the main idea is to use this general approach in a totally different context regarding people in order to discover and determine citizen movement flows. According to this definition, the general problem addressed in this paper focuses on achieving structured information about the movement habits of citizens in the geographical area of a city.

The main challenge to be resolved is the way in which the population can be traced preserving their anonymity, without necessarily requiring their collaboration, and in non-controlled and heterogeneous environments. In addition, the processing and centralization of information obtained must be considered to be useful for decision-making, and it should be aware that the number of citizens can be very large, even considering a small urban geographical area of an average city.

### 2.2. Literature Survey

The citizen track and trace issue is not new, as evidenced by the amount of research on this topic. Indeed, there is some concern about maintaining user privacy due to the proliferation of systems and high integration rate of mobile devices connected in the hands of citizens [16,17]. However, the benefit of knowing citizen’s traceability information for the design of cities has spurred studies and works which try to determine it while preserving the privacy of citizens.

Transport planning is an example of a task where an analysis of human and traffic flows can be very useful. This issue is essential since the populations of cities are increasing dramatically. Decisions regarding this have been studied for many years. As a result, there are numerous proposals for successful decision-making processes in transport planning [18,19,20,21]. Such decisions involve many actors such as members of the city council, representatives of the transport companies and even the citizens. Usually, council members along with the company representatives come together to form a regional consortium. Therefore, this consortium is the scenario where the decisions regarding public transportation are made. Setting the routes of the various services such as buses or subways is one the main decisions they have to perform. Due to this fact, they must analyse data about the city and its citizens in order to extract the knowledge about their habits and transportation necessities, *i.e.*, the analysis of human movement trends in urban areas. However, although these guidelines provide a well-studied framework for decision-making on transport, there are some critical shortcomings that have been detected in some of the current literature [22,23].

First of all, very often there is an issue of the obsolescence of design plans. Traditional planning processes can become obsolete when cities grow significantly. If too much time passes between when a project is planned and it is executed, by then it will be outdated and certainly will have lost some of its usefulness. To address this problem, there are works proposing a system based on the use of SC tools that allow projects to evolve dynamically with the possible changes in the scenarios over time [24].

Secondly, there is another important issue concerning the procedures used to get the data and especially how can they be used for providing decision support. Traditionally, the information used in these processes is obtained through field studies (e.g., manual recounts of people and vehicles, census, *etc.*) along with information obtained from interviews with the different actors involved, namely, citizens, transportation companies, and event managers, among others [22]. However, these massive surveys and interviews are very expensive, thus they cannot be performed frequently. Due to this fact, they rapidly become obsolete and if we trust such outdated information we will not take into account the real situation. Furthermore, these data sources lack information about individual trajectories and consequently we cannot compute the real individual movements of people [22,23], but rather we are able to obtain a global and a simple view of citizens’ movements.

It is highly desirable, therefore, to use technology to effectively determine the human traffic flows. The main technology created for this purpose is the Global Positioning System (GPS) [25]. With GPS, we can precisely and easily establish our position on the world map, so it is used for orientation purposes by drivers and pedestrians since it has become quite affordable. In addition, it is now integrated into many devices such as mobile phones or other ubiquitous devices [26].

An example of an application for this problem can be found in a study conducted in the cities of Milan (Italy) [27] and Beijing (China) [28]. In the case of the Italian city, the data come from GPS devices installed in a rental car fleet. In the Chinese city, the GPS devices were installed in a taxi fleet. These papers present some analytical methods as well as data mining tools in order to provide discovery knowledge processes. They rely on a central node responsible for processing all the information acquired from the GPS devices.

Based on the information obtained about the trajectories, there are several studies which focus on data analysis and manipulation to extract information of interest. In this regard, query languages and tools for trajectory analysis oriented to query trajectories computed from GPS devices have been established [29]. These researches develop a clustering technique that allows extracting traffic patterns over indicated periods of time. The results of these analyses allow one to trace road communication routes much faster than existing patterns and the results obtained by tools like Google or Bing maps.

However, the use of GPS raises some issues to consider. One of the most important of them is the transparency of use: access to the positioning data of users requires that they allow it, either by configuring or installing an application designed to carry out such data collection on their mobile devices. This configuration involves extra effort that not everyone may be willing or able to undertake. Moreover, if it is activated, the user will be aware that is transmitting data at all times. Therefore, it becomes clear that it is not a transparent mechanism for the user. In addition, there is an added problem: the preservation of privacy [30]. By knowing the exact location of people in real-time and the trajectories they have followed over time, it is possible to study their movement habits and even predict their location at a certain time of day. In the wrong hands, such information may compromise not only the privacy of citizens, but also their safety. For instance, it would be possible to know with precision at what a time a citizen is not at home in order to commit a theft. Finally, besides the two mentioned problems, an additional problem is the power consumption since devices require their own power source to operate in addition to the cost of the devices themselves. Despite being integrated into many medium and high range mobile phones, they are still relatively expensive if purchased separately.

The use of the installed wireless infrastructure, has also been considered for this application [31]. This includes mobile phone networks and wireless local (Wi-Fi) and wide (WiMax) area networks. These networks were not designed for this purpose but to rather access the Internet and communications services offered by telcos. However, from the moment in which each base station is aware of the presence and identity of each connected device, it is possible to offer tracking operations while the user is moving from one station to another [32,33]. Some research also includes filtering and interpolation operations to predict where the user is located [34,35,36]. However, due to their scope and current coverage, they cannot provide accurate situation information about where the user has passed. Moreover, placing a greater number of stations for this purpose could significantly increase the infrastructure cost.

Mobile telecommunications technologies (GSM, 3G, LTE, *etc.*), can also be used to perform large-scale tracking and tracing of users, however, they are not considered in this work due to the lack of necessary precision and the need of having a mobile terminal. Although their degree of penetration in the population is very high, their use for this purpose involves other drawbacks about cost and user privacy. Other alternatives such as CCTV, are discarded due to the technical difficulty of developing automatic methods for tracking individual users in uncontrolled contexts and the fact it can cause a stronger invasion of privacy due to the feeling of being “watched” by surveillance cameras.

An alternative to previous techniques for sensorization of citizen movements is to use Radio Frequency Identification (RFID) communication technology [37,38,39]. This technology has also been widely used to obtain location information of users and objects. Table 1 summarizes the main recent works on this issue as a representative sample. All the proposals analyzed work in controlled environments, that is, the infrastructure deployment has been designed previously and provided by the system managers. When the users access a system are given a tag to carry around the working environment.

Many studies have been conducted for track and trace indoors and outdoors. For instance, outdoors, the City of London introduced a system for data collection in its transportation system including buses, subways and trains [40]. The idea was to track users of the transport network through payment and access cards equipped with this communication technology. With the information gathered it was determined the level of use, number of travelers and public transport habits in the city and to know the stations, breakpoints, origin and destination of network travel streams. For indoors, systems to know citizen movement flows have also been implemented [41], for instance, visits per museum [42]. With the results, visit patterns of the different exhibits and areas were obtained, participants with atypical behaviours were identified and even relationships between visitors groups were established.

**Table 1:** RFID-based track and trace works.

Work	Main Application	Working Environment		Tag Deployment	Reader Deployment
		Indoors	Outdoors		
RFID enabled supply chains [11,12,13,14]	Traceability services	X	X	Passive tags embedded into objects	Fixed or mobile readers. Along supply chain
RFID delivery system [15]	Intelligent Transport Systems		X	Passive tags embedded into objects	Fixed or mobile readers. Along delivery system.
RTSV [43]	Tracking System for Vehicles		X	Passive tags installed on cars	Distributed throughout the city.
The London Oyster Card Data [40]	Public transport planning	X	X	Passive tags Inside transport cards	Installed on entries and exits of transport system.
iWalker [44]	Assistance services of location and obstacle detection	X		Placed anywhere in the environment	Embedded into walkers
Tracking science museum [42]	People Traceability	X		Passive tags on nameplates carried by users	Distributed throughout the floor
SIP-RLTS [45]	Location Tracking System	X		Passive tags carried by users (patients)	Readers carried by workers (medical)
LANDMARC [41,46]	Indoor location sensing	X		Active tags on grid array deployment	Distributed throughout the environment
Blind User [47]	Location and Proximity Sensing	X	X	Passive tags; Indoor: Grid array deployment over floor; Outdoor: Along edge of the sidewalk.	Readers carried by users
Pervasive mining [48]	Tracking people in a pervasive mining environment.	X		Passive tags carried by users	Distributed throughout the environment
Privacy-Preserving Solution [49]	Tracking People in Critical Environments	X	X	Passive tags carried by users	Distributed throughout the environment
Social interaction [50]	Person tracking	X		Passive tags carried by users	Distributed throughout the environment
Cameras and RFID [51,52]	Tracking and identification people	X		Passive tags carried by users	Distributed throughout the environment
RFID Inside [53]	Tracking and identification people	X	X	Passive tags inserted in users	Readers carried by workers (medical, security, *etc.*)
Tagging Demented Patients [54]	Tracking and identification people	X		Passive tags carried by users	Readers carried by workers (medical)
WSN and RFID [55]	Person tracking	X		Passive tags installed on objects or people	Distributed throughout the environment
Elderly Living Alone [56]	Person tracking	X		Passive tags carried by users	Distributed throughout the environment
Peer-to-Peer Networks [57]	Location Tracking System	X		Active tags carried by users	Distributed throughout the environment
REACT [58]	Children location		X	Passive tags carried by children	Distributed throughout the environment

RFID technology overcomes some of the disadvantages described for GPS, as it does not require too much cooperation from the user and reduces energy costs. However, its use for tracking does not provide exact locations but, like other wireless networks, the temporal location marks are limited by the locations of the signal reader antennas and the analysis of the Received Signal Strength Indicator (RSSI) of the readings [56]. Thereby the resolution of the trajectories followed by the users will be lower than in the case of the GPS. The outdoor scope of this technology is also not comparable to GPS coverage. There is a shorter range variation named Near-Field Communication (NFC) [59] that requires much less power to operate. Moreover, passive RFID/NFC technologies may be present in a multitude of objects and devices that people wear [38]. In many cases, they are integrated into standard cards made of plastic with identification and access functions [60], credit or debit cards [61,62,63] or even paper tags on clothing [64,65,66,67], however the functions for which these elements were designed were not tracking people in itself, but rather other applications such as localized identification, payment processes or object tracking in stores.

After the review carried out, it is observed that the activities of localization and tracking can provide valuable information to improve infrastructure development and user satisfaction for a variety of applications. From all the reviewed literature on track and trace systems, the most cited solutions show significant drawbacks. The Global Positioning System is the most suitable and it provides better results due to its precision and accuracy, however, this implies the necessary participation of citizens for track and trace activities. The citizen must have a device with computing and communication capability that enables it to provide the captured information, usually a mobile phone on which an application has been previously installed and configured. These requirements consume a considerable amount of energy that may affect the autonomy and the availability of the device. In addition, these solutions involve knowledge of personal data of the citizen like the terminal number. These features imply that the system is restricted to a limited set of the citizens, so the sample would be not representative to provide good service for managing the resources of all citizens. Elderly citizens who are not familiar with ICT, citizens who do not have access to such technology or citizens unwilling to provide personal data, among others, would not be represented in the datasets. Moreover, these proposals are only valid for open city environments, which would imply the inability to track citizens in indoor environments where GPS technology is not adequate. Nevertheless, these spaces also require efficient management of their resources.

To take advantage of the extensive set of connected mobile devices as well as the installed infrastructure, the use of wireless networks is another option in which proposals have already been made. However, the results it gets are not very precise and it also produces privacy intrusions as the identification of the user is known.

Another alternative that can be provided is the use of RFID/NFC, a technology increasingly found embedded in devices and other portable fixtures accepted by the public. For its functioning, not only energy consumption, but also expensive equipment, are not required. In this case the main obstacle is that the technology is underdeveloped and in general, antenna networks prepared for this purpose are not available.

Most related works that use RFID technologies are focused on tracking systems and only few proposals have contemplated both the track and trace problems. Most of them apply their solutions in closed and limited environments, where the characteristics of reliability, service quality and scalability are not necessary. However, the development and proliferation of this RFID technology among citizens during the last few years, opens up new possibilities for its use in track and/or trace tasks based on user-centric schemes. The characteristics of this technology may allow mixed deployment both indoors and outdoors. In the analysed work, the use of RFID technology is limited to reading the homogeneous tags provided by the system owner and sending information to a central system, without prior processing, storage or filtering, as these tasks are delegated to a central node, which can generate a bottleneck.

It is noteworthy that none of the literature proposals focus on the provision of the information collected to third parties, in a dynamic, personalized and open way. For this purpose, the development of cloud-based architectures may represent a good choice for the design of flexible management architectures and service providers. This aspect could enhance the development of decision support systems for the efficient management of resources and oriented to meet the needs of citizens. The scope of the limitations and advantages mentioned will be discussed in the following sections to lead to the design of the proposed architecture according to the objectives.

## 3. Research Methodology

The working hypothesis of this research is that it is possible to know and process citizens’ movement flows across the city by using new IoT technologies.

Once the problem has been described, the hypothesis established and the related work analysed, it is necessary that the comprehensive track and trace system becomes clearer. This system should allow the acquisition of locations of citizens in heterogeneous and uncontrolled environments, indoors and outdoors, to facilitate the transparent incorporation of citizens into the system. Furthermore, the information acquired should enable providing information about citizen movement flows to someone else for efficient management of the resources of cities and to be able to provide better services to these citizens.

In order to develop our proposal, a research methodology based on business process management [68,69] using the Eriksson-Penker formal notation [70] it has been followed. Process management is a strategy for structuring a complex process into a sequence of tasks that can be shown as actions that transform inputs into some other output elements. This transformation must be aligned with previously defined goals, which are considered as strategic, to meet some needs or gaps identified in the problem context. In this way, the defined process will achieve the object system of this work in a systematic way, selecting the most appropriate techniques and tools to meet the objectives in order to solve the identified problems. In the proposed methodology, each of the tasks identified represent a stage of the research, and therefore these will have associated one or more scientific methods as described below.

**Figure 1:**
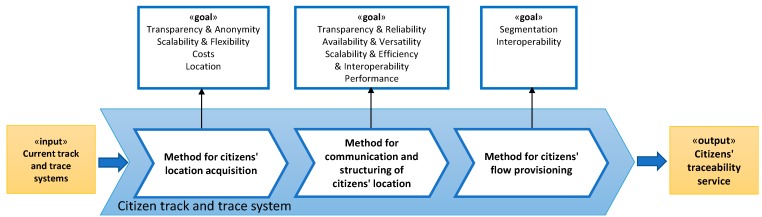
Modelling with Eriksson-Penker notation of the process of obtaining citizen movements flows.

Specifically, from the starting hypothesis, Figure 1 shows the main process carried out in the research, named *Design of citizen track and trace system*, where the input element («*input»*) represents current track and trace systems. This «*input»* must be transformed through this process into a novel citizen traceability service («*output»*), addressing the needs and gaps identified previously, which now are represented by the strategic objectives to be able to guide the research process («*goals»*). To perform the transformation to achieve the objectives established, the controllers («*Control»*) and facilitators («*supplies»*) which are required to guide this transformation must be identified. Particularly, these elements represent strategies, paradigms, techniques and technologies to be integrated into our proposal. In order to make easier its identification and, therefore, make clearer the problem, the main process has been divided into three sub processes or tasks.

The resulting sub-processes are the following (Figure 1): first of all, the sub-process *Design of the method for citizens’ location acquisition*, focused on establishing necessary technology infrastructures to get citizen data. Secondly, the sub-process *Design of the method of communication and structuring of citizens’ location*, involved in ensuring the correct reception of the citizen location data. Finally, the sub-process *Design of the method for citizens’ flow provisioning* focused on provisioning standard citizen flows to third parties.

**Figure 2:**
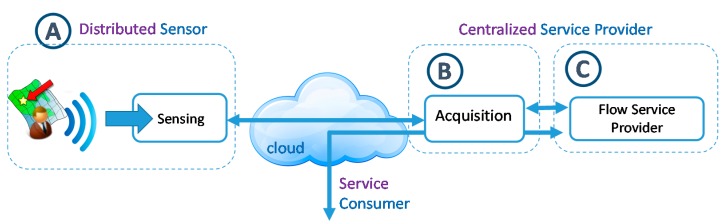
Overall Computational architecture of the citizen track and trace system.

The result of this process will be a computational architecture in order to read and process citizens’ movement following the overall scheme shown in Figure 2. The final design of this architecture will be led by the decisions made in each sub process of Figure 1. The research made, uses the knowledge and experience of the research group in the field of designing specialized computational architectures [71,72,73].

As can be seen, it is composed of a distributed module and a centralized one. The distributed module (A) implements the reading of user location data. The centralized module (B) acquires the data from sensors and provide to the consumers (C) the citizen traceability service to help when making decisions on urban management. The functioning and components of those modules will be detailed progressively in the next sections.

### 3.1. Method for Citizens’ Location Acquisition

**Figure 3:**
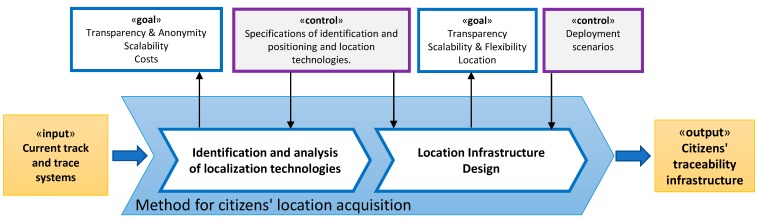
Modelling with Eriksson-Penker notation of Design of the method for citizens’ localization acquisition process.

This task is focused on the identification of the most suitable location technology and the location infrastructure design as a starting point for addressing the problem to achieve the following goals, as noted in Figure 1: gathering of geographic information (*location*) should be transparent to the citizen, meaning without requiring their cooperation and without their intervention in managing and setting the devices, without affecting its economy and independently of whether the citizens’ transits through both indoors and outdoors environments (*transparency*); the designed method will have to maintain the user anonymity and protect personal privacy, avoiding any capture of citizens’ personal and confidential information (*anonymity*); the technology used should allow its functioning and quality of service (QoS), regardless of the number of citizens interacting with it and the size of the area that will be monitorized (*scalability*) and the associated infrastructure should provide agile adaptation to any environment (*flexibility*); finally, the deployment of the necessary infrastructure should have a low financial and consumption costs (*costs*). With these design goals in mind the intention is to facilitate the placement of a valid infrastructure and minimize citizens’ rejection. Figure 3 shows a complete schematic of this process following the methodology criteria.

#### 3.1.1. Identification and Analysis of Localization Technologies

For the selection of localization technology, we take into account the analysis and the experience gained in the review of other work and the performance and benefits that are currently available [25,26,30,34,35,36,38,42,59,74,75,76,77].

Table 2 summarizes the main technical characteristics as they relate to the proposed objectives. Regarding the objective of transparency, obtaining positioning with GPS systems or Wi-Fi networks do not offer transparency and the desired anonymity for such applications, since in both cases the collaboration as well as users’ permission is required. Specifically, it is necessary that a citizen has a device that supports these technologies, he must install and configure an application designed for this purpose, provide the permission to transmit location information, which may include personal information, and, for all this, he must have a minimum knowledge and technological skill. In addition, the accuracy and coverage of GPS could be a privacy and legality concern, in terms of providing more than the location information necessary for the purpose of the proposal, which is the efficient management of city resources. Besides, although other wireless technologies (Wi-Fi, GSM, *etc.*) could determine the location, the accuracy to address the problem described would not be valid in all cases. All this could hinder the acceptance of the proposal by the general population and therefore it would not be possible to obtain representative samples for the established purpose.

**Table 2:** Main features of communication technologies.

Technology	GPS	RFID/NFC	Wireless Networks
*Main architecture*	Triangulation positioning by satellite.	Set of antennas or readers and receivers.	Set of antennas or readers and receivers.
*Communication*	The user receiver obtains the signal from satellites and calculates the position.	Readers inspect receivers to determine whether they are present.	Receivers report that they are present.
*Operating frequency*	1100 MHz to 1600 MHz	Active: 455 MHz, 2.45 GHz, 5.8 GHz; Passive: 128 KHz, 13.6 MHz, 915 MHz, 2.45 GHz	Wi-Fi: 2.4 GHz, 5 GHz; WiMax: 2.3 GHz, 3.5 GHz; Cellular mobile: 800 MHz, 1900 MHz, others.
*Cover*	Worldwide; Outdoor environment	Depending on antenna network deployed. Outdoor and indoor environments	Depending on antenna network deployed. Outdoor and indoor environments.
*Range*	Worldwide	Active: ~100 m; Passive: 0 to few meters	Wi-Fi: 30 to 100 m WiMax: ~50 km; Cellular mobile: ~35 km
*Power consumption*	Very high	Passive tags receiver: Very low.	Very high
*Deployment Costs*	Satellites: Already deployed and free to use; Present in mobile devices	Need to deploy readers network. Present in mobile devices and other everyday user accessories.	Need to deploy antenna network. Present in mobile devices and other user accessories.
*Localization*	Receiver position.	Antenna position.	Antenna position.
*Transparency and Anonymity*	Low	High	Low
*Usual application*	Navigation, topography, land levelling, *etc.*	Identification, access control, payment, *etc.*	Internet access and communication services.

It is true that in terms of cost or ease of deployment of the infrastructure, there is already a high degree of integration of devices with GPS or Wi-Fi connectivity among the population. However, the energy cost of their use is high and its use may only for track and trace reasons not be justified. Finally, all the technologies are scalable to the deployment of infrastructure used, although the GPS positioning has an advantage since it does not require additional elements apart from the satellites. However, in the domain of the problem studied, the use of GPS is limited in indoor areas which should also be analyzed to manage their resources.

For all these reasons, among the analysed alternatives our proposal is to use RFID communication technology to track and trace citizens. As mentioned in the previous section, these technologies are having an expanding and continuous implementation and currently many devices incorporate it [78]. This allows the use for this purpose of the tag park that is becoming larger and it is currently already installed in many applications such as payment cards [63], clothing [64,65,66,67], access to transport services [40,62] or tourism [60].

In most previous applications the user’s identity is not known and therefore it transmits only the identifier of the RFID tag worn by the user (in most cases, the tag could be attached to interchangeable everyday elements such as an umbrella or book which could be carried by different users). Consequently, the system doesn’t know the correspondence between citizens and RFID tags. This technology does not allow a user to refrain from providing the location, since he or she will not be able to tear off the RFID tags from their clothes, accessories or cards. In that sense, using this technology for traceability should be treated similarly to CCTV which already has appropriate legislation: Deployed coverage infrastructure can be limited to specific areas, both indoors and outdoors and always inform the user about it, *etc.* However, there are also methods to preserve anonymity with RFID technology [53,79,80] which can be implemented in open city environments. In addition, the system may reduce the acquisition of unnecessary information which might involve intrusion into user privacy.

This technology provides installation options with passive or active receivers according to the desired range and complexity of the receiver. In the case of passive receivers, no power supply is required for transmitters [76,77], which reduces the drawbacks of consumption and portability for the user and enables its use for extended periods of time.

Regarding the economic cost of the proposal, the utilization of existing tags among the population reduces the cost of deployment. In this aspect, the physical nature of communication technology enables the use of metal urban furniture items as card reader antennas [38]. In this way, elements such as street lights, billboards, traffic signs or traffic lights can all operate as antennae. In addition, most of them are connected to the network, and then they can reduce the cost of the necessary elements for the information transmission towards the central system.

Finally, in terms of the ability for concurrent reading, the selected technology is able to work with a high level of traffic to simultaneously capture the location of a large number of tags located in the antenna reading radius. For example, the standard reader used in the experiments has a throughput up to 400 concurrent readings per second [81].

#### 3.1.2. Location Infrastructure Design

The RFID technology mentioned in the previous section consists of two types of elements: The antennae or readers that emit the signals and receivers or tags. As mentioned, in the passive mode of this technology, receivers do not require power and can be embedded in objects familiar to users such as keychains, bracelets, keys or plastic cards worn by users.

Although this technology was conceived for identification processes, nowadays, it is also used for location tasks. Concerning this topic, research works are focused on the design of reader networks where each of them has associated geographical coordinates. Thus, from the received readings and deployed topology, it is possible to determine the position of the users (*location*).

**Figure 4:**
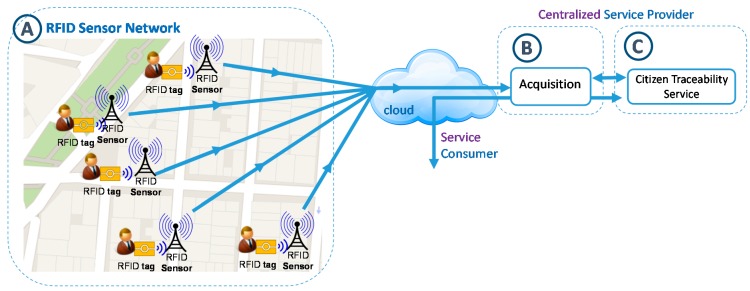
RFID sensor network deployment.

The aim of this process is to design a network of RFID sensors deployed throughout the urban area of interest for reading the locations of citizens (e.g., shops, streets, avenues, parks, government buildings, *etc.*). The advantages associated with this technique make it easy to adapt to heterogeneous contexts (*flexibility*), and both indoor and outdoor environments (*transparency*). Moreover, adding new readers to the network is feasible according needs of the urban area management (*scalability*). The next figure (Figure 4) schematically shows the A-part of the overall architecture such a network of RFID sensors deployment in a city environment.

**Figure 5:**
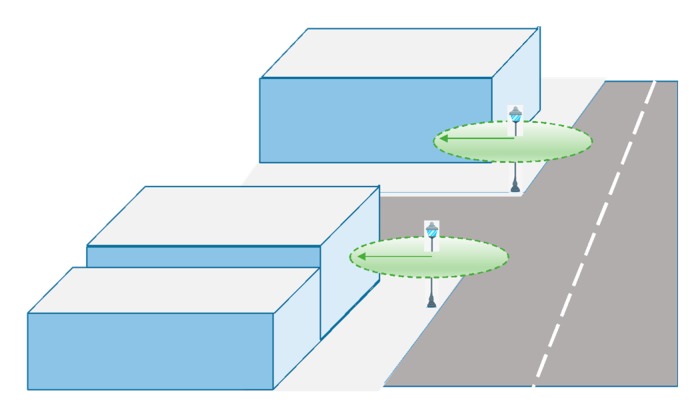
Installation and sensor coverage example.

According to the features of RFID technology, it is possible to configure readers with coverage radii of several meters [76,77], so each of these readers can cover the sidewalk of a street entirely, as shown in Figure 5. The purpose is that collaboration of citizens for the activation of tags is not required and all they have to do is allow that they be worn on their clothes, carried in a pocket, bag or wallet in a transparent manner and interact as they move without the user being aware of it. Thereby, the tags worn by citizens are activated when they walk down the street and pass by one of the installed antennas.

The grid topology must be dense enough to cover the circulation in areas of interest. For practical reasons, tags used must be in some application that is already widespread and accepted by the citizens, while as mentioned, the new antennas can be complemented by already installed infrastructure.

With regards to the deployment of this additional infrastructure various criteria may be taken into account. For example, for practical criteria it may be decided to cover only areas of special interest in the city (downtown, tourist areas, *etc.*) or for economic criteria it may be decided to limit the grid to some areas of the city. In this regard, there may be options for installation of mobile antennae that can move from one part to another of the city when the area is sufficiently studied.

### 3.2. Method for Communication and Structuring of Citizens’ Location

In the analyzed works based on RFID technology, the proposed solutions are focused on restricted areas, such as supermarkets, homes, hospitals, logistics centers, places of entertainment, *etc.* where the information transmission is performed directly through private intranets (not on public networks like the Internet) without reliability capabilities. In these proposals only an RFID tag type is used and processing needs have not been considered. However, after this analysis, it was identified that one of the challenges of the proposed system was the heterogeneity and lack of environmental control where it is mandatory to act in order to acquire the citizens’ location information to build the movement flows. In this sense, any space in the city can be improved, without knowing the number or the type of tags to read or the capacity limits of the required processing. In this new scenario, it is necessary to propose a system with the technology and infrastructure indicated in the previous section, capable of distributing the logic of the system to ensure the workload processing, transmission and storage and structuring of information can be carried. This will establish the citizen flow and, therefore, the objectives will be addressed as outlined in the new scenario.

This is the objective of the task described in this section. More specifically, the goals which these process should cover are: To be capable of acquiring and processing the information independently of environment or data structure of the RFID tags (*transparency*), regardless of the number of readings performed by the park of sensors deployed in the network (*scalability*); to ensure the delivery and reception of each location to generate the different citizen flows (*reliability*) without citizen identification data (*privacy*); to ensure that the information is accessible from any place and on any device for the service consumers (*availability and versatility*); to offer complete information about the citizen movements, despite the lack of certain individual locations (*completeness*); to manage the systems resources in an optimal way (*efficiency*) and to distribute the load (*performance*), due to the high amount of location information that every reader will be able to receive and send to the central system; and, finally, to be interoperable in order to obtain information from different location formats or methods (*interoperability*), as the system is intended to take advantage of the already deployed elements and incorporate them for users who could might work with different protocols or formats.

**Figure 6:**
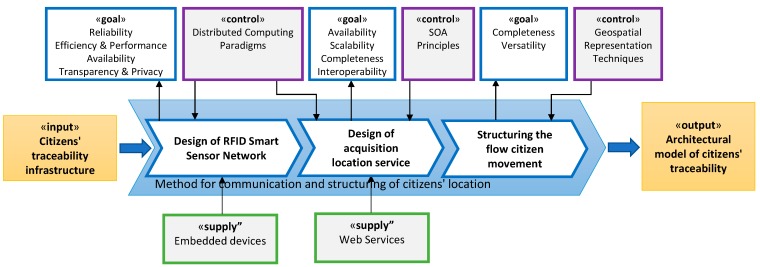
Modelling with Eriksson-Penker notation of the details of the process *Design of the method for communication and structuring of citizens’ location*.

In this sense, an analysis of techniques, patterns and design principles that guide and control the transformation process to achieve the proposed system and reach the mentioned goals has been necessary. The result of the process is a distributed architectural model based on the use of RFID Smart Sensors networks whose functionality will solve the problems (A-part of overall architecture), and a centralized cloud-based system which complements the information acquisition features. The RFID Smart Sensors basically consist of an RFID reader with computing and communication capabilities. The centralized cloud-based system will focus on the generation (B-part of overall architecture) and provisioning of citizens’ flow (C-part of overall architecture) in an open and transparent way. The result of this process presents a reference model based on the integration of patterns and software design techniques. The novelty of this method lies in its combination and slight modifications to address the underlying problem and to solve existing deficiencies in current systems. This combination satisfies the use of RFID technology in uncontrolled and heterogeneous environments for determining the flow of citizens’ movement in order to provide an efficient management of the resources of the cities. Figure 6 describes the processes involved in it, according to the methodology used with its main goals.

Next, we will describe the specifications of these elements in order to achieve the information acquisition, structuring, and communication goals.

#### 3.2.1. Design of RFID Smart Sensor Network

**Figure 7:**
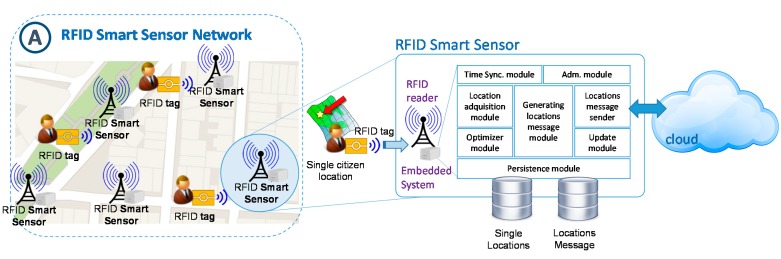
RFID smart sensor architecture.

Although there are related works which propose distributed architectural models based on sensors network to get the individual locations of the citizens (for example [42,45,48,50,53,56,58], these approaches do not cover necessary aspects (reliability, privacy, performance, efficiency, availability and transparency) to give a solution to the problem described. One of the main contributions and novelties of this paper is the provision of intelligence to sensors. This device is mainly responsible for reading the locations and ensuring the delivery of such information. So that, in order to provide the right route generation from each movement, it is necessary to design the infrastructure as a network of RFID Smart Sensors which include the necessary rationality to track citizens. Therefore, each Smart Sensor consists of an RFID reader, together with an element of embedded processing that provides computing and communication capabilities to support the necessary functionalities to reach established goals. These functionalities are supported by eight modules such as is shown in the schematic architecture of the RFID Smart Sensor (Figure 7).

One of the main functionalities included in the RFID Smart sensors is the capacity to receive information from different RFID tags types, transparently. About this issue, only the proposal presented in [45] proposes a solution that allows the capture of the location information from various types of RFID tags through the use of ontologies. Although this proposal would require a prior knowledge of the structure of information stored in the RFID tag to be read, an aspect that could mean that the management of ontology is untenable. It should be necessary to know all the existing tags types or every time is detected a new one to introduce the information into the ontology.

The remaining works analysed can only capture tag information provided by themselves. Since it is required in the problem proposed, our solution is not dependent on the RFID tag. In the Smart Sensor proposed, it has included functionality to obtain, transparently, full information as mentioned, from the generation of a code from the information that has been read to identify the location of form unique. When a new acquisition of the same tag arrives, the same code would allow traceability is created. Although a security system is included to increase the privacy of citizens that is every day changes the code generation. There is no wish to know if it is the same person carrying the tag, but the traceability of all citizens. These functionalities are performed by the *Location acquisition module* of the Smart Sensor described in Figure 7.

To perform reliability issue, we will implement the integration pattern known as guaranteed delivery [82]. This pattern determines an architectural design which prevents the loss of information messages transferred among the computer nodes that make up a distributed system. The pattern is based on the previous persistent message storage from a node in local disk. Until the destination node does not confirm message reception and storage, the source node will not delete the message from its local storage system. If the destination node never confirms message reception, the source node would retry sending it. Only with the use of the mentioned pattern, the complete delivery of the data cannot be ensured. The data could be sent and, because of the existence of communication problems, message reception confirmation may not arrive at the RFID station. In this case, if the message is not transmitted, the data would not be deleted and there would be a loss of information; or if the message were resent, data would be duplicated. To solve this issue, we focus on the correlation identifier pattern [82]. Each submission message will be marked with an identifier formed by a time stamp, which can establish the correlation for further processing, and by the identifier of the source RFID reader. Once communication is restored, the message would be sent again, being the acquisition module in the server the responsible for verifying information duplication. This way, we can ensure the complete reception of the necessary information to infer citizen flow patterns without losing or duplicating messages. This is another novelty functionality achieved through of patterns integration included in the RFID smart sensors. These functionalities and patterns are implemented by *persistent module, location message sender* and *update module* of the Smart Sensor shown in Figure 7.

Another key aspect to ensure delivery reliability in a distributed system, and it is not contemplated by the rest of the research works that provide RFID sensors distributed architectures, is the time synchronization of the system components. Most of the analysed proposals are focused on the tracking process [45,46,47,48,49,50,51,52,53,54,55,56,57,58]. The proposals focused on traceability process do not contemplate this question [42]. However, this aspect is necessary to achieve the tracing process. In order to generate reliable routes to manage resources in a better way, it is necessary to know the specific moment of the citizen location. A device provided with a badly synchronized clock would be sending the location at a wrong moment, the route information would not be valid, and it could even lead to wrong decisions. There are many algorithms that solve the problem of time synchronization, and choosing a particular one depends on the required accuracy of the system, the resources to be used, and the information that we want to obtain. For the present proposal, we will reject those systems that work with relative times and logical clocks, because, in our case, it is essential to synchronize all the elements of the system with a UTC reference system.

Depending on the type of network used and the precision required, we will have a great number of methods, among which are classical solutions such as Network Time Protocol (NTP), Simple Network Time Protocol (SNTP), and Time Transmit Protocol (TTP), or more advanced solutions such as Reference Broadcast Synchronization (RBS), Time Protocol on Sensor Networks (TPSN), and Flooding Time Synchronization Protocol (FTSP) [83,84].

In our case, the abovementioned patterns, together with time synchronization, ensure the reliability in the data delivery process. Also, the precision of the synchronization may perfectly tolerate a few-second lag, as the route would be relative to the local level and a range of several RFID readers would not appreciate such a lag. Moreover, once the server receives the location information from several neighbouring readers, there could be a data optimization and standardization regarding time. In the RFID Smart Sensor, this functionality is performed by the *time synchronization module*. This module is an autonomous and independent component that is executing periodically in background to update the clock system (Figure 7). In addition, the design proposed includes the following considerations in order to reduce the communication and management costs of the RFID smart sensor: Firstly, it is necessary to achieve an optimal transmission rate to take advantage of the bandwidth, which will be determined by the communication protocol established in the sensor. If we send the information via HTTP, the submitted location information could be less than the message wrapper of the communication protocol used. This circumstance would trigger penalties in each message sent. In our proposal, the number of locations in each sent message would be configurable in the Smart Sensor in order to achieve the best forwarding rate depending on the transport protocol used and the available bandwidth. The RFID Smart Sensor includes an *administration module* to manage and parameterized remotely the network sensors; secondly, the storage capacity of the Smart Sensors is limited, so it is essential to minimize local storage. In this sense, there would be a small modification of the guaranteed delivery pattern, in which not only would the message be erased to get the reception confirmation, but also the location data that were included in the message. With this design, once the RFID smart sensor obtains the location of a citizen, it stores it locally, creates a message from the citizen location data which is registered in the database, and stores them again. When the message is sent to the information system of citizen flow and is stored, the system returns reception confirmation to the client and the message is finally erased from the local system. This process is performed by the *update module* of the RFID smart sensor.

The remaining of the research works performs the communication process of the location data in controlled environments regardless of the communication reliability. For this reason, they do not propose the persistent capability at the sensor level and they have not contemplated the resources optimization of the sensors as our proposal.

Below, the proposed design works according to the following procedure: as the RFID reader is reading different citizen tags, these are received by the local location acquisition device through the acquisition module that performs the role of Coordinator.

Whenever an RFID tag content is received, the timestamp (only year/month/day) is attached to it and then the hash function is applied. The result of this action will be used to perform anonymity to the citizen. Then, this information is sent to the *optimizer module*, which is responsible for indicating if a citizen location must be stored or discarded depending if it is considered redundant data or not. If the information is valid, the *acquisition module* stores it in the local location database (*persistent module*) and informs the *generating location message module*, which uses the data to control if it reaches the optimal number of acquired locations to create a message that will be stored in the location message database. When the *location message sender* detects that there are new messages, it begins to transmit them by connecting to the *citizen flow generation service or acquisition location service*. The message will send the data through the available transport protocol.

At this moment, the RFID smart sensor will wait to the acknowledgement message location from *citizen flow generation service*. When the sensor receives it, then its update module is invoked, deleting the corresponding message and their individual locations from the database in the same transaction. This operation allows optimizing resources. If during a time previously configured the message acknowledgement was not received, the location message sender would return to try to send the message, as many times as was configured the device parameters. Before, the correct operation of network communications has to be checked.

#### 3.2.2. Design of Acquisition Location Service

This service must provide an interface for data acquisition, which allows receiving location messages from users of smart sensors ensuring scalability and availability of the system, the reliability in data delivery and the interoperability with different types of sensors. This service corresponds with B-part of the overall architecture. The following figure shows the internal details of this module in conjunction with the smart sensor.

**Figure 8:**
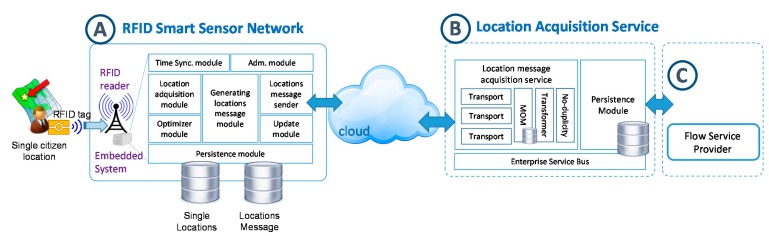
Location acquisition service architecture.

Scalability will be determined by two factors: firstly, the number of citizens who could interact with the system could grow indefinitely and, therefore, the data size to be acquired by the system could also grow in a reliable way. Secondly, the number of sensors deployed for the acquisition of citizen information may be high and could also grow considerably in order to obtain more data and to ensure accuracy in the flow determination. These two factors could make the citizen flow service provider become a bottleneck when it comes to receiving and processing the information. In order to avoid these situations, we propose two solutions that have been implemented: The first solution would be to separate the process of receiving and storing location messages, which requires less processing, from the transformation and processing of location data included in the messages. This solution would be provided by the message oriented middleware (MOM) paradigm or the software asynchronous queuing design pattern [85] (MOM component in Figure 8), establishing a point-to-point model based on message queues as an interface to the *location acquisition service*. This is a distributed computing model, which is asynchronous and transactional, and which acts as an intermediary message in the communication between a sender, the RFID Smart Sensor, and a consumer, the *citizen flow service provider*. In addition, this pattern enables the messages to remain, allowing us to implement the abovementioned guaranteed delivery pattern to ensure the reliability of the message communication; the second solution, which is complementary to the previous one, consists of the introduction of clustering techniques for load balancing using multiple nodes to be distributed and placing a messaging system for data acquisition in each of these nodes. Thus, the burden of data acquisition would be fairly distributed, and even the implementation of new acquisition nodes would be immediate.

Although the use of MOM paradigm has been proposed in [45] to provide scalability in indoor tracking system processing, in this proposal the communication reliability between sensors and central system and fault tolerance of the central system are not contemplated. Moreover, no aspect of high availability and load balancing is contemplated on this functionality as the needs that require scalability and processing are limited by the application environment.

In relation to the availability, a centralized system collapse or failure may involve the loss of location data and, therefore, reduces the validity of the generation of citizen flow patterns. To solve this problem, clustering techniques could be added to the acquisition module in order to achieve high availability. This way, if one of the nodes falls, another one from the cluster would replace it.

Finally, another important issue to be considered when location data are sent from RFID smart sensors is the heterogeneity of the existing client devices in the market and the communication protocols they use to transmit data. There are RFID readers which have computer systems that allow communication through standards which are known and widely extended. However, others only include serial communication and a small low-cost embedded device should be included to transmit information. In addition, although the proposal mainly relies on collecting and acquiring citizen locations through RFID technology, the system could consider acquiring data through other types of location and positioning systems such as GPS or legacy data systems that provide location information. In turn, this would lead a diversity of message formats and data models.

**Figure 9:**
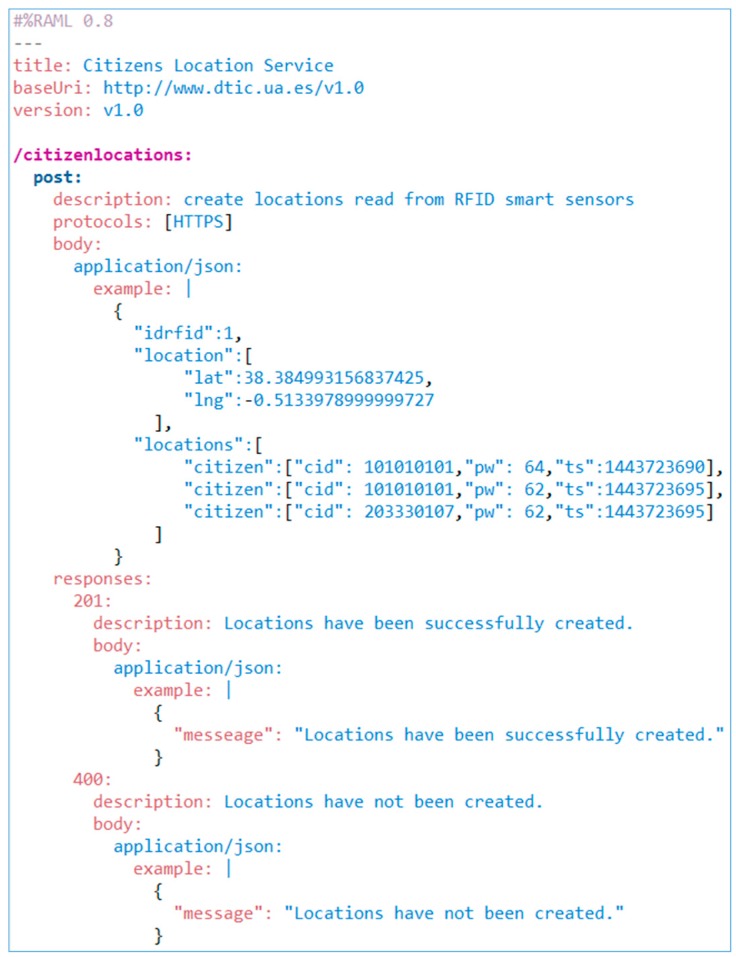
RAML contract of location message acquisition service.

In those cases, we have considered creating an infrastructure based on protocol bridging patterns, data model transformation and data format transformation [85]. The protocol bridging design pattern is implemented by different transports modules (Figure 8), although, by default, the transport module of the system described in this section is based on Rest style following the SOA paradigm. The Rest service contract has been defined by means of *Rest APIs Modelling Language*, RAML (Figure 9). The other two patterns are implemented by the *transformer* component shown in Figure 8. The goal of the transformers is to unify the message in order to be the performance of citizen traceability service provider. In this way, we can dynamically solve the heterogeneity problems of the message structure, the data format and the communication protocol used for respectively sending the acquisition module.

When the message is received through the correct transport module, the message is stored in a persistent management system (point to point queue messaging model based on the pattern *asynchronous queuing* [85]). Messages are stored waiting to be consumed and processed. From this moment, the system sends an acknowledgement message to the RFID smart sensor to confirm location message reception.

It could happen that the message had been received by the system, but the acknowledgement response sent by the system fails because the communication was interrupted. In this case, a message previously processed could be processed another time. This is the function of the *no-duplicity* module, to filter the messages and to avoid their reprocessing using the message identifiers (message_id and rfid_reader_id). When a message is received from the queue of the message system (MOM), their structure and data format is checked. Depending on this information could be possible the use of transformers (*data model* and *format transformation* patterns) [85].

All modules of the location acquisition module are supported by *Enterprise Service Bus* (ESB) platform. ESB is a powerful infrastructure for software integration and communication that enables the interoperability between the described modules.

#### 3.2.3. Structuring the Citizen Movement Flow

From the requirements analysis performed in the preceding paragraphs, in this process we must identify the information required to ensure the generation of reliable citizen flows which will allow making decisions in the management of the city resources. In order to do so, the main goals are the provision of complete information concerning the geographical area under study (*completeness*), with the possibility of segmenting the information depending on the time of day, the day of the week and the time of year (*versatility*).

The most basic and essential entity in order to determine the citizen flow of a citizen, will be the location (L). This entity will be defined by the following tuple:

L ≡ {*rfss* (*lo*, *la*), *cid*, *pw*, *ts*}

where:

• *rfss* (*lo*, *la*) represents the location of the RFID Smart Sensor determined by its longitude and latitude, which will allow us to know the position of the citizen on the map.
• *cid* represents the radio-frequency identification which is carried by the citizens and which allows us to identify them in a unique and anonymous way.
• *pw* it is the power of a certain card reading signal which will allow us to know the proximity to the RFID Smart Sensor antenna. Based on this, the information on the RFID Smart Sensor will be preprocessed in order to eliminate redundant locations with no added value which belong to the same user and will help determine possible directions of a citizen or trajectory changes with the data from other neighbouring RFID Smart sensor.
• *ts* it is the moment in time in which the RFID tag reading took place.

This data will be sent to the centralized system by the Smart Sensor, following the mechanisms described above to ensure delivery. The other data entity needed to determine the citizen flow is the deployed RFID smart sensor network itself. This information, together with the individual locations, will be completed to determine the route taken by a citizen. The sensor network entity will be determined by the following elements:

*RFSSN ≡ {RFSS*_1_, *RFSS*_2_,…., *RFSS*_n_*}*

*RFSS*_k_*≡ {id*_k_, *lo*_k_, *la*_k_, *N*_k_*}*

*N*_k_*≡ {RFSS*_i_…*RFSS*_j_*}⊂RFSSN*

where:

• *RFSSN* represents the set of RFID Smart Sensors that make up the sensor network.
• *RFSS*_k_ is a specific sensor defined by its unique identifier (*id*_k_), its position, determined by the longitude (*lo*_k_) and latitude (*la*_k_), and the set of its neighbouring smart sensors (*N*_k_), which in turn will be a subset of the network (note that the geographical-neighbours of a sensor could not match with its real neighbours due to insurmountable obstacles or habits of movement of citizens).

The data received by the deployed sensor network should be structured and completed in order to build the citizen movement routes in the geographic area under study. Therefore, knowing the neighbouring sensors allows addressing potential locations losses on the route followed by a citizen and helps define the interpolation and completeness strategies according to methods known to do so.

The combination of the data collected will be used to generate on-demand citizen flows for specific criteria, such as time interval or geographical area, indicating their density or the possibility to foresee citizen movement flows according to behaviour patterns based on historical movement data.

### 3.3. Method for Provisioning Citizen Flow

**Figure 10:**
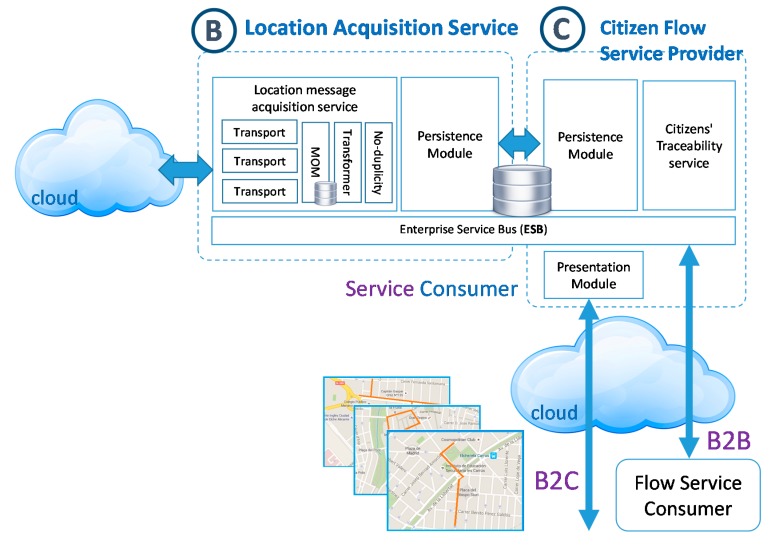
Location acquisition service architecture.

The orientation of this research to the study of public spaces in the city has led us to design methods to provide the information generated openly and as a value added service. In this section existing technological paradigms are used to enable easy consumption by third parties. The next figure (Figure 10) depicts schematically this service (C-part of overall architecture) in conjunction with the rest of the centralized architecture.

According to the foreseen, the goals focus, firstly, in the on-demand supply of the citizen movement flows based on the needs and search criteria for consumers (*segmentation*). Secondly, it is important that the value-added service can be used by external applications (*interoperability*). Therefore, this task has been divided in two stages: The design of data search filters and the supply of the system as an interoperable service.

In the first stage, several filters have been established to search based on the consumer needs. The first filter would be aimed at determining the action area where we want to make the decision. This action area could be defined by different indicators with a greater or lesser degree of accuracy such as a specific location, a specific location and a radius of action, a specific route, an area previously defined by the urban policy makers, or, finally, some irregular area determined by the service user. The second filter would determine the time period about which we want to obtain citizen flow data for the specific area and which would be given by parameters that represent different precision levels, from a short period of time in the same day to a long period in the long term, which could be months or years. Another filter would be based on the density of routes, allowing the information about specific routes that exceed or are below a threshold and that can contribute to the modification of the use of city resources. For example, a route with low density for a long period of time may lead to the modification of a public transport route.

**Figure 11:**
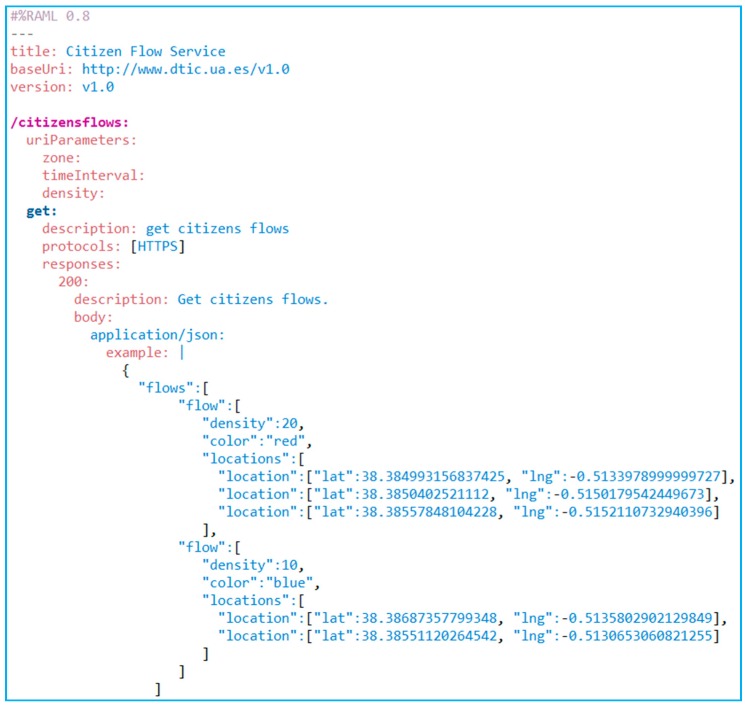
Citizen flow service RAML contract.

As mentioned before, the second stage focuses on securing the interoperability of the service with the consumers. In order to do so, this service should offer the following two models: A first approach based on Business to Business (B2B) paradigm will allow connecting the consumer system with the service to incorporate it as another one of its organizational processes, but improving the current system by adding more decision data. Both cases will be based on the service supply according to the principles of the Service Oriented Architecture (SOA) paradigm, [85] implementing through Rest service technology and offering the resulting information using a format that is structured as JSON, so that it is easily treated. The service contract, modelling with RAML, is shown below (Figure 11). As can be seen, the service contract includes the filters defined previously to carry out searches by means of next parameters: Action zone, time period and flow density; a second approach, based on the Business to Customer (B2C) paradigm, oriented to offer a human-machine interface which will allow a user to interact directly with the system, for example through a thin client such as a Web browser. The access interface of this module defines the search filters described previously. When the Rest service receives these parameters, this one returns the citizens flows and their density drawn on cartographic map. In both cases, the citizen’ flows will be generated from the information collected by RFID Smart Sensors and the information of the sensors topology grid dynamically and on demand, based on the input parameters representing the filters entered by the consumer. The generation of flows combined through individual routes of the same or multiple users may be caused by adding readings for the specific filters through a clustering process.

The calculation of these added routes can be performed through several procedures by applying a similarity function to them. For example, some of the functions of this type used are the comparison of the common points of the sequence, and the comparison of distances or times used in them. These procedures are beyond the scope that defines this architecture, although they are being considered in the line of work developed by the research group.

## 4. Implementation and Validation

### 4.1. Implementation

**Figure 12:**
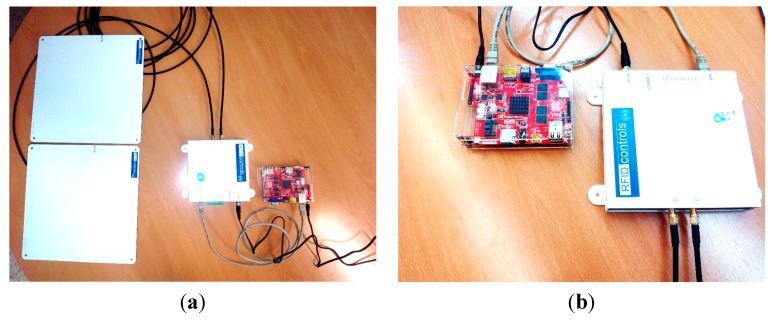
RFID Smart Sensor prototype: (**a**) RFID Smart Sensor with antennas; (**b**) RFID reader with an embedded system.

In order to be able to validate the proposal, a prototype of the architectural model shown previously has been implemented. The RFID Smart Sensor prototype is shown in Figure 12. The sensor used is marketed by *RFID Controls Enterprise* (Murcia, Spain). This is a highly configurable and flexible sensor with two antennas [81]. It has a maximum reading rate of 400 tags/s and its maximum reading potency is 1 W. This device even allows connection to two antennas through two 50 Ohm MCX connectors with 6.5 dB gain, providing circular polarization and a radiation pattern with 60°/60° beamwidth (Figure 12a).

In order to provide the sensor with the necessary intelligence for implementing the aforementioned modules, an embedded device based on an ARM processor has been used. This device has a low energy consumption and it has been integrated with the RFID reader through its Ethernet interface. Specifically, the Cubieboard3 cortex-A7 dual-core 2 Gb embedded device has been used, provided by *Cubietruck Enterprises* [86] (Figure 12).

The RFID Smart Sensor modules designed in the architectural model have been implemented using C language. Reading results are saved in CSV files and stored in a system directory. To implement the clock synchronization module the NTP protocol has been used, because is a reliable protocol with suitable error rates for our system. For this reason, an NFS server has been enabled, which is used to update the system clock by means of the synchronization module implemented in C language.

**Figure 13:**
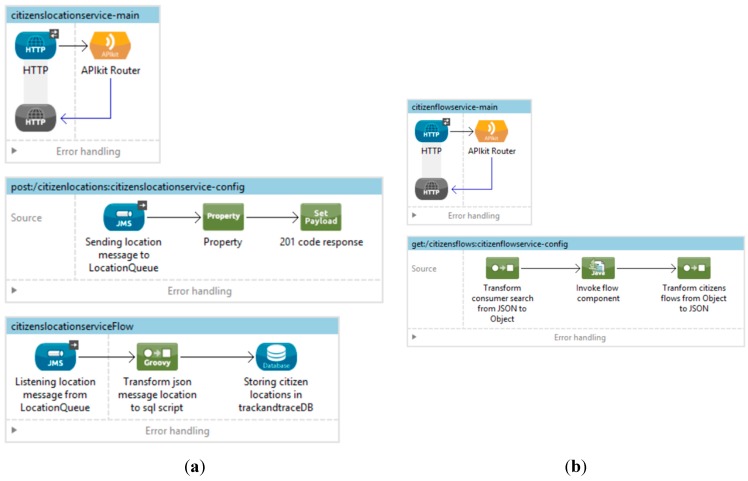
Mule flow. (**a**) Location message acquisition service; (**b**) Citizens flows generation service.

The proposed architecture of the citizen track and trace system is based on *Enterprise Service Bus integration infrastructure*. This solution is highly scalable and the software design patterns specified in the architectural model have been implemented over this platform. Specifically, the open source Mule ESB has been used, implemented two Mule ESB services flows to implement the *Location Message Acquisition Service* and the *Flow Generation Service*, shown in Figure 13a,b, respectively.

In order to provide data persistence, the *MySQL community version* database manager has been used. The *Search Module* for flow generation has been implemented using Java Language. This module is embedded and invokes the Mule service flow. Finally, the *B2C Presentation Module* has been developed through Web technologies like CSS, HTML5 and JavaScript using the AngularJS framework, in order to provide more accessibility and usability to the final users.

In order to achieve better analysis and interpretation of the experimentation results obtained in the case study described below, we have previously made a technical testing of the smart sensor and the RFID technology. The aim is to know beforehand their capabilities and limitations to certain situations that may occur.

First, we proved that with the power supplied by the sensor (1 W) and with the well-focused antenna the reader is capable of reading tags up to 10 meters away. Secondly, reading tests were performed simulating actual portability by citizens, *i.e.*, placing tags hidden in clothes, pockets, purses, handbags or backpacks. These experiments significantly reduce the sensor range down to 3 m, but a good reading rate is still maintained in these cases.

**Figure 14:**
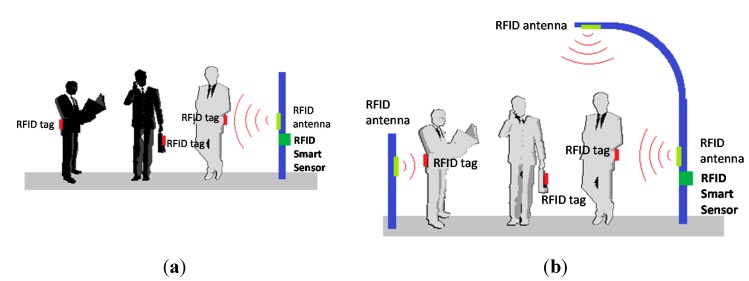
Configuration of the antennas and coverage of RFID sensors: (**a**) One antenna on the right side of a sidewalk; (**b**) Three antennas on the right, left and top of a sidewalk.

The most serious problem encountered for our purpose is that the human body is opaque to the RF signal, so that the tag will be hidden from the sensor when there is a human obstacle. This case can be common in urban settings with a high density of pedestrians. For example, in Figure 14a, the two persons on the left are in a shadow area produced by another person. However, this problem can be solved by distributing the antennas of the smart sensor in a configuration that minimizes occlusions and signal shadow areas. Following the previous example, Figure 14b shows a configuration that reduces the space not visible to the sensor.

Regarding the other capabilities of the Smart sensor, the experiments proved the ability to synchronize with other sensors, the combination of multiple readings from the same person and the capability of sending information without loss. Through log systems of the proposal of the traceability of reading, we analyse sent and received citizens’ locations and we verify that all locations readings are stored in the *RFID Smart Sensors Network* and they were received by *Location Acquisition Service*. In this way, the reliability of the proposed architecture is validated.

### 4.2. Case Study

In order to validate our proposal as well as to study a practical implementation of the concepts treated, a simulated use case in a real environment for tracking and tracing is proposed in this subsection. In our example, we consider the campus of the University of Alicante as a test scenario for the deployment of the proposed system. The case study will provide a better understanding of how various campus resources are used based on student and staff spatial movement and temporal behaviour. The ultimate goal of this case is to help the university improve management of the support services on campus.

The experiments performed will also result in a number of spatial and temporal visualizations of big data from these sources. The reason to carry out a project on a campus is the advantage of disposing of something similar to a little city, but more accessible. In a way, since it has different areas (rooms, labs, small shops, restaurants, open areas, *etc.*), diverse activities (academic, administrative, social, small health centres, sports…) the university campus can be seen as a small city. In addition, it has the facilities to make it as a perfect candidate to test our system under diverse conditions in order to find out citizen movement flow patterns. It is always far less complicated to locate antennas, RFID readers and the rest of technology within the university campus than throughout a city. In addition, it is possible to monitor the different states of every single location point, allowing testing of what are the best and most suitable places for the whole system. Therefore, in the matter of fact, we can see our system implemented in the framework of the campus as a prior step for a smart city which is much bigger, although it can be benefit from this previous stage.

The criterion for placement of the readers has been to monitor the most likely movements of the students (cafeteria-classrooms, classrooms-library, bus station-classrooms, *etc.*) as a preliminary step to a more dense deployment. Students were not informed that they were taking part in this experiment, only academic campus authorities were informed to request the appropriate permissions for the deployment of the readers. Due to this, no tags were distributed among individuals, but our intention was to first validate the transparency and anonymity of the readings, the ability to read tags and carried by people (on clothing, tickets, identification or transport cards, other RFID tags) without their cooperation and the capacities of transmission and processing of the architecture.

The experiment was conducted during one week. Through a visual monitoring of the antennas the number of students who passed through the sensor radius was counted. It was about 2K per day, with a tag read rate of about 5%. The amount of simultaneous readings of this data remained low, although, in most cases, when the tags were read by an antenna, they were read again by the next antenna on the same route.

From the study of these results, the reasons that lead the low rate of readings obtained can be: (1) the still low level of integration of RFID tags on clothing and other objects carried by users; (2) the lack of power in the readers to reach tags hidden in the wallet in the pocket of the user; and (3) the incompatibility of technologies with short-range tags (as NFC) to access ID cards or credit cards.

As to the first drawback (1), we think that will be resolved eventually, as the steadily increasing pace of implementation of RFID technology in everyday objects such as clothes or accessories will increase the reading rate. The second problem (2) can be overcome using directed antennas with a low angle of beamwidth coverage and high gain antennas rather than general purpose ones since in outdoor spaces, the signal can be dispersed more or noise sources can even appear [87,88]. In this regard, it is necessary to check regional regulations to find out the maximum allowable radiated power and antenna beamwidth in the UHF RFID bands. Finally, regarding the third cause (3), we think that this kind of RF technology belongs to a set of applications that should not be accessible, so to remain inaccessible, reinforcing anonymity of identity and user data.

In all cases, the readings collected and sent to the server correspond to a tag identifier on clothing or an accessory. This information consists, among others, of the following fields: An ID tag of different length, IP address of the reader, received signal power and DateTime (*i.e.*, 30396062c3c324800009e6 77, 169.254.247.184, −74, 03/02/2015 08:55:59.121). Under no circumstances is personal user information collected. Due to the functionality included in the *Location Acquisition Module* which codifies RFID tags content through hash functions, we could not get any data of those RFID tags. In addition, any of the read RFID tags could be related themselves between readings obtained from different days.

The central system is hosted on a server on the campus intranet. The network traffic generated and the system processing times are trivial for a standard workstation, as the number of simultaneous readings remained low all the time, reaching traffic peaks of up to 3 reading/s.

**Figure 15:**
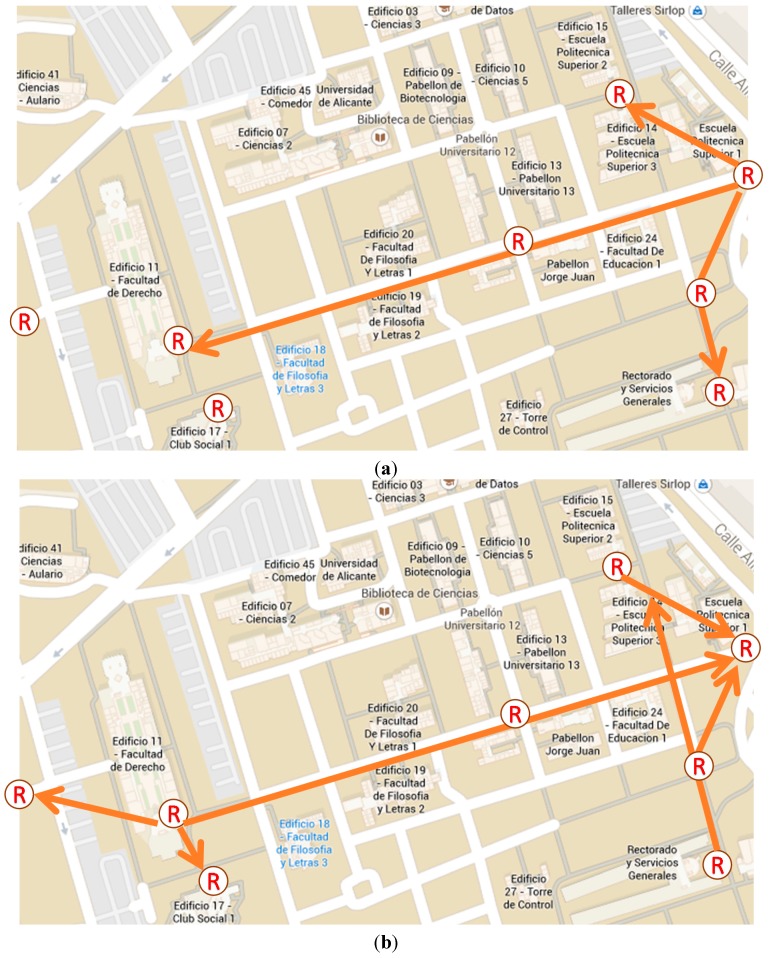
Temporal visualizations on the University Alicante campus with flow patterns: (**a**) temporal flow between 7.00 and 9.00 h; (**b**) temporal flow between 12.00 and 14.00 h

To view the results we used a web-based user interface, which shows the positions of the readers and users-route through a third party application (Google Maps JavaScript API v3). For this case study, the next figure (Figure 15) shows a map of the University of Alicante showing the installation locations of the reader antennas and two temporal visualizations along the day where one can distinguish the main flow pattern per hour.

The results show that the movement flows of students change throughout the day. In Figure 15a it can be seen that the antenna located near the tram and bus platform receives students first, and from there, they are read by sensors located in other areas of the university as they move around the campus, both via inbound and outbound scenarios. In these hours of the morning, the movements are primarily targeted towards classroom buildings, offices and the library. Figure 15b shows the movements at other times of the day, when students walk to the campus exit and to restaurants and cafes to have lunch.

In this case study, the students’ movements read by the system may seem obvious at first glance, since they coincide with the normal movement at that time of day. However, this case study provides the starting point for further deployment in which the movements of students and staff of the university can be analyzed in more detail. This information can help managers to provide better services and improve user satisfaction by providing resources where they are needed at any given moment.

In light of these results, we can assert that this experiment verifies the validity of the proposed hypothesis regarding the capabilities of RFID sensing technologies to read the positions of citizens outdoors without needing any kind of collaboration. The correction of distributed architecture to collect, synchronize and to process readings received while maintaining the anonymity of the users involved, it is also validated. The workload has not been a problem in this context due to the low rate of simultaneous readings. It is expected that a larger-scale deployment will allow us to validate the system in this regard. This result provides a promising perspective in the development of IoT for interaction with users.

Finally, a summary of the main objectives achieved by the proposed architecture in comparison with the capabilities of the other proposals is shown in Table 3. The comparison is focused on the results obtained in the experimentation, indicating what objectives would be achieved with each proposal. The table illustrates that related works offer partial solutions to complete all the stated objectives.

**Table 3:** Comparison of the obtained results between our proposal and the related works.

Research Work	Main Functionality	Transparency & Anonymity	Working Environments	Reliability	Energy Consumption	Scalability
Proposed architecture	Track & trace	Yes. No user interaction & Independency of RFID tags	Indoor & outdoor	Yes	No (passive tags)	Yes. (Distributed and decoupled approach)
GPS proposals [27,28,29,30]	Track & trace	No. User interaction	Outdoor	No	High	No
Wi-Fi proposals [16,34]	Track	No. User interaction	Indoor & outdoor	No	High	No
CCTV	Surveillance	No. Surveillance cameras	Indoor & outdoor	No	High	Yes
Science museum [42]	Track & trace	No. User/Object interaction & Dependency of own RFID tags	Indoor	No	No (passive tags)	No
Oyster Card [40]	Track & trace	No. Dependency of own RFID tags	Indoor & outdoor	No	No (passive tags)	No
Blind/inside RFID [47,53]	Track	No. User interaction & Dependency of own RFID tags	Indoor & outdoor	No	No (passive tags)	No
REACT [58]	Track	No. User/Object interaction & Dependency of own RFID tags	Outdoor	No	No (passive tags)	Yes
LANDMARC [41,46]	Track	No. User/Object interaction & Dependency of own RFID tags	Indoor	No	Yes (active tags)	No
Peer-to-Peer Networks [57]	Track	No. User/Object interaction & Dependency of own RFID tags	Indoor	No	Yes (active tags)	Yes
iWalker [44]	Track	No. User/Object interaction & Dependency of own RFID tags	Indoor	No	No (passive tags)	No
Other [45,48,50,51,52,54,55,56]	Track	No. User/Object interaction & Dependency of own RFID tags	Indoor	No	No (passive tags)	In controlled env. only [45,55]

## 5. Conclusions

The design of smart cities uses the information generated in the city itself for decision making aimed at improving the quality of life of citizens. Infrastructure planning is one of the most pressing challenges of the development of cities and usually the handling of this issue does not only correspond to practical criteria, but also social, political, historical or other aspects. Knowledge of citizens’ flow movements is very valuable information for designing transportation routes, as well as the optimal positioning of resources. With this knowledge, it is feasible to perform infrastructure designs which can minimize deviations from real movement flows. It is also possible to increase transport dynamic strategies in accordance with these needs to meet the movement habits of people by time of day, day of week or time of year to increase the use of infrastructure and thus improve the comfort of the citizens. The latest ICT advances can offer promising solutions to this specific problem by providing efficient and effective ways for handling large amounts of unstandardized and distributed information.

The proposal described in this paper is the design of an architecture that can read, process and provide as a service the movements of individuals in a given geographic area based on real-time information acquired from the individuals themselves as they move along the city in an unassisted way to improve the resources management of cities.

The research presented in this paper includes an exhaustive analysis of the state of the art of techniques for solving the citizen traceability problem in cities. Its conclusions have allowed us to identify limitations of the current methods and technologies on the basis of the following main criteria: Heterogeneity, maintaining anonymity, no user intervention, uncontrol and cost. A comprehensive architectural model has been conceived using RFID wireless technologies with passive tags present in many devices, accessories or the clothes of the citizens to perform track and tracing tasks in city environments. As a novelty, we have designed an RFID smart sensor to read, process and to send the information of the users’ locations in a scalable and reliable way to ensure the delivery and reception of each location. Finally, we have developed a computational architecture which integrates all the elements and modules implied to provide cloud services of citizens’ traceability for B2B and B2C, consumers based on SOA principles.

The way of achieving of goals established and solution of problems raised have been the main contributions of this research. The solution has been in most cases to identify techniques and patterns that partially solve these issues to integrate them and offer a global solution. It is worth mentioning that the integration has brought changes in patterns and adjusting these elements to offer a comprehensive solution we have not found in the literature reviewed. In addition, these solutions have been encapsulated in functions and distributed in elements building the architecture to ensure the requirements of the problem. As a result, a network of intelligent sensors is provided, which is a novel solution for the current track and trace systems, fully coordinated and a central system is added to provide the citizens’ flow.

The work involved in this research fits into the concept of Smart City aimed at improving lives of citizens using current technology of communications, cloud computing and smart sensing. In this regard, some challenges for future work arise, such as combining other communication technologies (GPS, Wi-Fi, *etc.*), integrating other sensing devices to improve monitoring (smart phones, *etc.*), studying mechanisms to optimize the network, new sensors deployed to cover the largest area possible or applying the presented architecture in a real city scenario. Moreover, the combination of these communication technologies with social media data such as Twitter and Facebook, will help develop broader patterns of citizen behaviour and compromise, service and facility utilization.
